# Operative vs non-operative management of ventral hernia: a population based study of long-term benefits and consequences

**DOI:** 10.1007/s10029-026-03845-x

**Published:** 2026-09-16

**Authors:** Sourav Kumar Podder, Julia A Baran, Scott H Koeneman, Francesco Palazzo, Nicole L Dugan, Christopher Keating, Sami Tannouri

**Affiliations:** 1 Department of Surgery, Thomas Jefferson University Hospital, 1015 Walnut Street Curtis Building Suite 613, Philadelphia, PA 19107 USA; 2 Sidney Kimmel Medical College, Thomas Jefferson University, Philadelphia, PA USA; 3 Department of Physical Therapy, Thomas Jefferson University Hospital, Philadelphia, PA USA; 4 Department of Physical Therapy, Thomas Jefferson University, Philadelphia, PA USA

**Keywords:** Ventral Hernia, Abdominal Core Health, Physical Therapy, Regional Interdependence, Ventral Hernia Repair

## Abstract

**Purpose:**

Ventral hernia (VH) management has progressed beyond simply physical repair of the defect. It now involves a strategy that purposefully restores abdominal core function and prevents future complications. This study investigates the association between ventral hernia repair (VHR) and functional disorders related to the abdominal core in addition to bowel obstruction, opioid misuse, and physical therapy utilization.

**Methods:**

This retrospective cohort study used administrative codes from the TriNetX database. The outcomes of interest were spine and pelvic floor dysfunction, constipation, incontinence, genital prolapse, bowel obstruction, opioid misuse, and physical therapy utilization.

**Results:**

A total of 587 384 patients were identified with diagnosis of VH. Of those, 81 830 (13.9%) underwent surgical repair. Surgery was associated with a significant reduction in the hazard for bowel obstruction (HR 0.65, 95% CI [0.63, 0.67]). There were no association between surgery and the hazard of opioid misuse (0.97, [0.93, 1.01]). The hazard ratios associated with surgery were 1.05 (1.02, 1.07) for spine and pelvic dysfunction, 1.03 (0.99, 1.05) for constipation, 0.97 (0.93, 1.01) for incontinence, and 0.88 (0.72, 1.07) for genital prolapse. The hazard ratio for physical therapy utilization after surgery was 1.76 (1.73, 1.80).

**Conclusions:**

VHR is associated with reduction in the hazard of bowel obstruction, while having no significant effect on opioid misuse. Additionally, surgical patients had a higher utilization of physical therapy. While surgical management was not associated with a reduction in the hazard of functional disorders, further research is warranted to better understand these relationships.

**Supplementary Information:**

The online version contains supplementary material available at https://doi.org/10.1007/s10029-026-03845-x.

## Introduction

Ventral hernia repair (VHR) is among the most performed surgeries worldwide, with more than 600 000 repairs annually in the United States alone [1, 2]. The spectrum of ventral hernia (VH) symptoms spans from asymptomatic presentations to life-threatening complications including bowel obstruction [3]. The primary goals of VHR have traditionally focused on relief of pain, the prevention of intestinal complications such as bowel obstruction, while reducing the risk of hernia recurrence [4].

Abdominal Core Health (ACH) is an emerging concept that is defined by the function, stability, and quality of life (QoL) associated with the abdominal core. The abdominal core is comprised of the circumferential soft tissues of the diaphragm superiorly, the pelvic floor inferiorly, and the abdominal wall and flank anteriorly and laterally. This schema posits that the abdominal core is integral to a wide range of physical activities, from sitting and standing to more complex actions like twisting, exercising, and performing essential bodily functions like urination, defecation, and respiration [5, 6].

Utilizing this holistic framework, VH management has progressed beyond simple physical repair of the hernia defect. It now involves a comprehensive strategy that ideally restores the long-term and complete functionality of the abdominal core as an integrated functional unit, improves the patient’s QoL, and prevents future complications. While the concept of ACH has been introduced in the greater hernia-related discourse, there is limited evidence on how VHR affects core health holistically, particularly in long-term function, stability, and QoL [5–9].

The goal of this study is to explore the population-level association between VHR and functional outcomes related to the abdominal core, including spine and pelvic dysfunction, constipation, incontinence, and genital prolapse, as well as bowel obstruction, opioid misuse and physical therapy utilization. By characterizing this knowledge gap, we hope to inform future clinical practices and develop a more comprehensive approach to VHR management both technically and perioperatively.

This study investigates association between elective VHR and patient outcomes compared to non-operative management regarding the new onset of spine and pelvic dysfunction, constipation, incontinence, genital prolapse, bowel obstruction, opioid misuse, and physical therapy utilization.

## Methods

### Data source

This retrospective, population-based cohort study used deidentified data from TriNetX (TriNetX LLC, Cambridge, MA). TriNetx is a global health-collaborative, clinical-research platform that collects real-time data from electronic medical records from over 90 healthcare organizations across the world. The TriNetX platform includes diverse patient data from over 100 million individuals. TriNetX provides access to curated, structured data which includes demographics, diagnoses, procedures, medications, lab results, tumor characteristics, oncology treatments, and genomics using coding systems such as International Statistical Classification of Diseases and Related Health Problems, Ninth Revision (ICD-9), Tenth Revision (ICD-10), International Classification of Diseases for Oncology (ICD-O), Common Procedural Terminology (CPT), Logical Observation Identifiers Names and Codes (LOINC), etc. Direct access to individual patient charts and clinical notes are not available. This study was evaluated by the Thomas Jefferson University Institutional Review Board and deemed exempted as it utilized only deidentified population-level records.

## Study population

This study utilized diagnostic and procedure codes from ICD-9, ICD-10, and CPT to define the two cohorts (Supplemental Digital Content 1). The non-operative cohort was comprised of individuals with VH without surgical repair after diagnosis. The operative cohort was comprised of individuals with VH and subsequent elective surgical repair between 5 and 365 days of VH diagnosis. We included all ventral hernia sizes and complexities, comprising of both primary and incisional hernias, and all types of elective repairs, including open and minimally invasive approaches. We excluded any patients that had surgery within 5 days as these were assumed to be emergent cases and not elective. We excluded VH diagnoses associated with coding for “obstruction” or “gangrene,” as well as VHR procedure codes indicating “incarcerated or strangulated” hernias, since these more often require urgent or emergent surgery. Patients with parastomal hernias and patients under 18 years of age were also excluded.

## Outcomes and covariates

The post operative outcomes of interest were diagnosis of bowel obstruction, opioid misuse, physical therapy utilization, in addition to functional outcomes such as spine and pelvic floor dysfunction, constipation, incontinence, and genital prolapse. ICD-9 and ICD-10 codes were used to determine outcomes of interest (Supplemental Digital Content 2). These outcomes were assessed following the diagnosis of VH, indicating that they occurred after the diagnosis. Confounding factors we queried were obesity, diabetes, nicotine-dependence, chronic obstructive pulmonary disorder, hypertension, cancer diagnosis, and congenital malformations and deformations (Supplemental Digital Content 3) [10, 11]. Congenital malformations and deformities (ICD-9/10 chapter Q00–Q99) were included as a covariate because several conditions within this broad diagnostic category — including connective tissue disorders, abdominal wall developmental anomalies, and other musculoskeletal malformations — have been associated with both an increased predisposition to VH formation and baseline differences in abdominal core function [12, 13]. We acknowledge that this ICD chapter is clinically heterogeneous and captures conditions with widely varying relevance to abdominal core health; it was therefore included as a broad comorbidity-adjustment variable rather than a specific marker of abdominal wall pathology. Its similar prevalence in both cohorts (7.2% vs. 7.2%, Table 1) suggests it was well balanced between groups and unlikely to be a major source of confounding in this analysis. Additional variables collected included age, sex, race, and regional location in the US, which included Northeast, West, South, Midwest, and outside US.

**Table 1:** Demographic and Clinical Characteristics of Patients with Ventral Hernia in the Time-Varying Analysis (*N* = 587 384)

	Total Cohort *N* = 587 384	Surgery Cohort *N* = 81 830	Non-Surgery Cohort *N* = 505 554
Demographics			
Age (years), median (IQR)	50 (40, 59)	50 (40, 58)	50 (40, 59)
Sex			
Male	286 976 (51.0)	42 419 (54.5)	244 557 (50.4)
Female	275 974 (49.0)	35 352 (45.5)	240 622 (49.6)
Race			
American Indian or Alaska Native	2019 (0.3)	276 (0.3)	1743 (0.3)
Asian	8500 (1.4)	865 (1.1)	7635 (1.5)
Black or African American	86 164 (14.7)	9514 (11.6)	76 650 (15.2)
Native Hawaiian or Other Pacific Islander	2080 (0.4)	266 (0.3)	1814 (0.4)
White	377 590 (64.3)	57 958 (70.8)	319 632 (63.2)
Other	27 638 (4.7)	3568 (4.3)	24 070 (4.8)
Unknown	83 393 (14.2)	9383 (11.5)	74 010 (14.6)
Location			
Midwest	74 157 (12.6)	13 776 (16.8)	60 381 (11.9)
Northeast	205 279 (34.9)	27 685 (33.8)	177 594 (35.1)
South	205 716 (35.0)	27 866 (34.1)	17 7850 (35.2)
West	63 602 (10.8)	10 930 (13.4)	52 672 (10.4)
Outside USA	20 794 (3.5)	248 (0.3)	20 546 (4.1)
Unknown	17 836 (3.0)	1325 (1.6)	16 511 (3.3)
Medical History			
Obesity	159 486 (27.2)	19 880 (24.3)	139 606 (27.6)
Diabetes	100 835 (17.2)	10 809 (13.2)	90 026 (17.8)
Nicotine Dependence	30 429 (5.2)	4383 (5.4)	26 046 (5.2)
Chronic Obstructive Pulmonary Disorder	34 779 (5.9)	3725 (4.6)	31 054 (6.1)
Hypertension	215 745 (36.7)	27 620 (33.8)	188 125 (37.2)
Cancer Diagnosis	67 318 (11.5)	9062 (11.1)	58 256 (11.5)
Congenital Malformations	42 381 (7.2)	5878 (7.2)	36 503 (7.2)
Outcomes			
Perioperative			
Bowel Obstruction	59 693 (10.2)	17 331 (21.2)	42 362 (8.4)
Opioid Misuse	29 311 (5.0)	4954 (6.1)	24 357 (4.8)
Physical Therapy Utilization	63 089 (10.7)	13 328 (16.3)	49 761 (9.8)
Functional			
Spine and Pelvic Floor Dysfunction	93 952 (16.0)	14 840 (18.1)	79 112 (15.6)
Constipation	60,465 (10.3)	9 808 (12.0)	50 657 (10.0)
Incontinence	23 150 (3.9)	3506 (4.3)	19 644 (3.9)
Genital Prolapse	1036 (0.2)	189 (0.2)	847 (0.2)

Values in parentheses are percentages unless indicated otherwise

### Statistical analysis

An analysis was performed using the above-mentioned covariates to look at the estimated association between surgery and the hazard of each outcome of interest. A Cox proportional hazards model was developed to evaluate the time from diagnosis of VH to each outcome. Surgical status was included as a time-varying covariate and was assigned a value of 0 prior to surgery and 1 afterward. This approach ensured that patients were appropriately categorized based on their surgical status during each time-period and reduced immortal time bias. In each model, we included age, as a non-linear spline term, sex, obesity, diabetes, nicotine dependence, chronic obstructive pulmonary disorder, hypertension, cancer, and congenital malformations and deformities.

Given the exploratory nature of this analysis, p-values are not reported, as no pre-specified adjustment for multiple comparisons was applied [14]. To address missing data, a complete case analysis was performed, including only patients with fully observed data for all predictors and outcomes. Consequently, individuals were included in each Cox proportional hazards model only if they had no missing values across the relevant variables.

All analyses were performed using R version 4.3.1 (R Foundation for Statistical Computing, Vienna, Austria).

## Results

### Demographics and unadjusted results

A total of 587 384 patients were identified with diagnosis of VH, with a median age of 50 and 275 974 (49.0%) being a female. Missing data was identified in 24,434 patients and these were excluded in our analysis. Of the included patients, 81 830 (13.9%) underwent surgical repair. The surgical cohort had a slightly higher proportion of males compared to the non-surgical group (54.5% vs. 50.4%). Most of the patients in both cohorts identified as White (70.8% in the surgical group vs. 63.2% in the non-surgical group), followed by Black or African American individuals (11.6% vs. 15.2%, respectively). Racial distributions across other groups were similar between cohorts **(**Table 1).

Comorbidities were prevalent across both groups. Compared to non-surgical patients, those who underwent surgery were less likely to have obesity (24.3% vs. 27.6%), diabetes (13.2% vs. 17.8%), COPD (4.6% vs. 6.1%), hypertension (33.8% vs. 37.2%), and cancer diagnosis (11.1% vs. 11.5%). Surgical patients had a slightly higher rate of nicotine dependence (5.4% vs. 5.2%). Both cohorts had a similar rate of congenital malformations (7.2% vs. 7.2%) **(**Table 1).

Perioperative outcomes showed that bowel obstruction was more frequently documented in the surgical group compared to the non-surgical group (21.2% vs. 8.4%). Similarly, physical therapy utilization (16.3% vs. 9.8%) and opioid misuse (6.1% vs. 4.8%) were higher in surgical patients.

With respect to functional outcomes, spine and pelvic floor dysfunction was more prevalent among surgical patients (18.1% vs. 15.6%) as was constipation (12.0% vs. 10.0%). Rates of incontinence and genital prolapse were comparable across groups (Table 1).

## Time-varying analysis

**Fig. 1:**
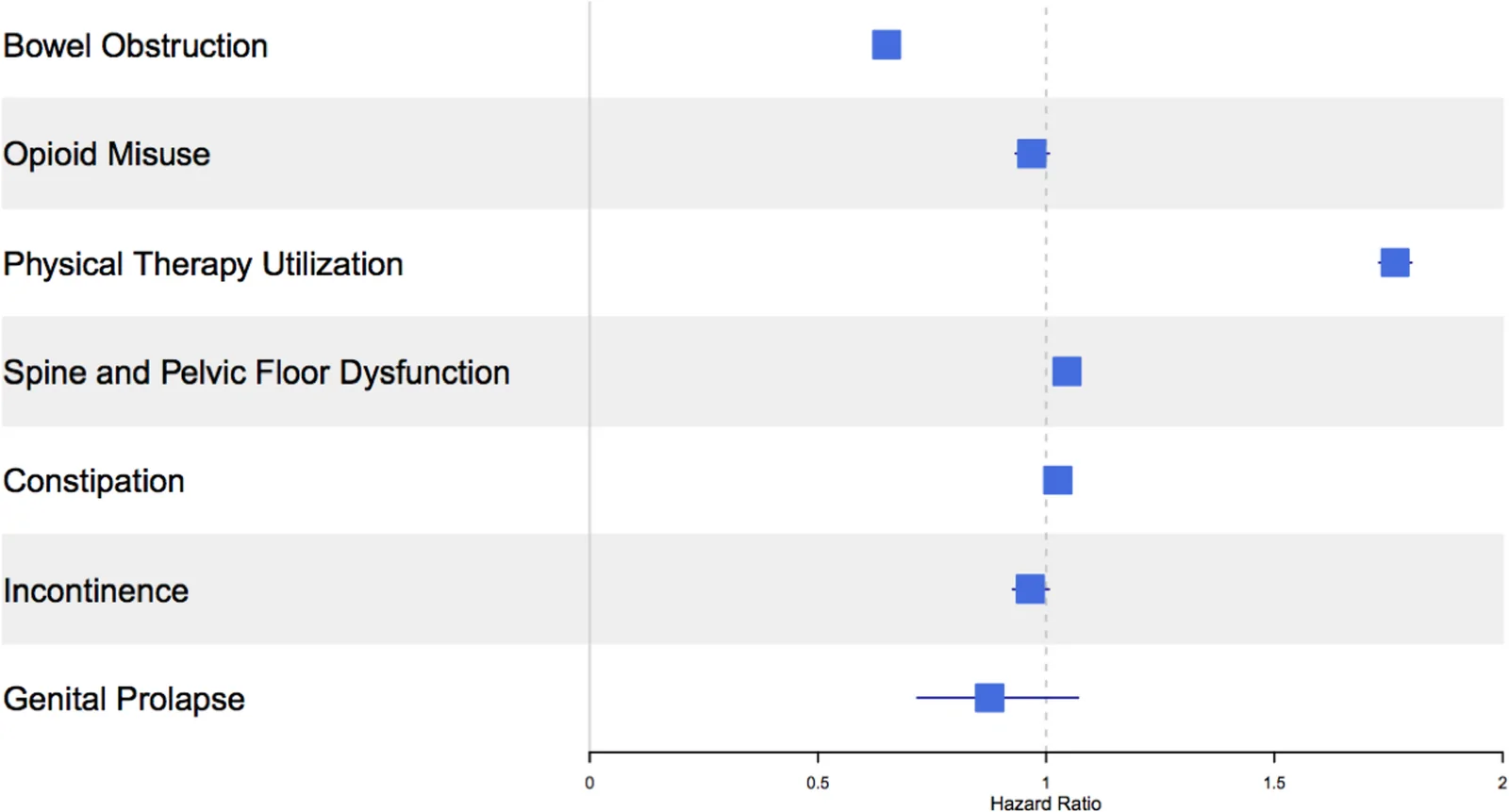
Model-Adjusted Hazard Ratios of Outcomes of Patients with Ventral Hernia Repair as a Time-Varying Covariate

Surgery was associated with a significant reduction in the hazard for bowel obstruction (HR 0.65, 95% CI [0.63, 0.67]). There were no substantial changes estimated in the hazard of opioid misuse (HR 0.97, 95% CI [0.93, 1.01]) after surgery. Conversely, the hazard of physical therapy utilization after surgery was significantly higher (HR 1.76, 95% CI [1.73, 1.80]) (Table 2; Fig. 1).↑

**Table 2:** Model-Adjusted Hazard Ratios of Outcomes of Patients with Ventral Hernia as a Time-Varying Covariate

	Hazard Ratio	95% CI	Summary
Perioperative Outcomes			
Bowel Obstruction	0.65	0.63–0.67	↓
Opioid Misuse	0.97	0.93–1.01	=
Physical Therapy Utilization	1.76	1.73–1.80	↑
Functional Outcomes			
Spine and Pelvic Floor Dysfunction	1.05	1.02–1.07	↑
Constipation	1.03	0.99–1.05	=
Incontinence	0.97	0.93–1.01	=
Genital Prolapse	0.88	0.72–1.07	=

*CI* Confidence Interval

For functional diagnoses the hazard for spine and pelvic dysfunction was higher among the surgical cohort (HR 1.05, 95% CI [1.02, 1.07]). In contrast, the hazard of constipation (HR 1.03, 95% CI [0.99, 1.05]), incontinence (HR 0.97, 95% CI [0.93, 1.01]), and genital prolapse (HR 0.88, 95% CI [0.72, 1.07]) were similar in the surgical cohort compared to the non-operative cohort (Table 2; Fig. 1).

## Discussion

This analysis demonstrates that elective VHR is associated with a reduced hazard of bowel obstruction. However, it was not associated with a significant change in the hazard of opioid misuse. Furthermore, surgery showed no meaningful association with the hazard of functional disorders including constipation, incontinence, and genital prolapse, while slightly increasing the hazard of spine and pelvic dysfunction. Lastly, surgical patients were found to have a higher hazard of physical therapy utilization compared to non-surgical patients.

These findings suggest a nuanced picture regarding the outcomes after VHR. While VHR was associated with a lower hazard of bowel obstruction, its association with other outcomes such as opioid misuse, constipation, incontinence, and genital prolapse were limited. Nevertheless, these outcomes might not be entirely independent of surgical intervention, and more targeted measures, along with prospective study designs, could better clarify the true impact of surgery.

Bowel obstruction is a major concern in patients with VH due to the risk for intestinal strangulation and ischemia [3]. In this study, although the raw proportion of bowel obstruction was higher in the surgical cohort (21.2% vs. 8.4%) (Table 1), the time-varying analysis showed that surgical repair was associated with significantly lower hazard of bowel obstruction (HR 0.65, 95% CI [0.63, 0.67]) (Table 2; Fig. 1). This discrepancy between raw proportions and hazard ratio estimates is due to differences in follow-up time (821 days in the surgical cohort vs. 562 days in the non-surgical cohort) and the analytic approach. Because patients were censored at the time of loss to follow-up, the hazard ratio estimation accounts for varying observation times. Additionally, in the time-varying analysis, patients were classified based on their status at the time of the event. Among patients who experienced both surgery and obstruction, approximately 81% had the obstruction occur before surgery or concurrent to the surgery and were considered part of the non-surgical group in the time-varying analysis. This approach was applied to all other outcomes as well.

This finding aligns with the understanding that repairing the hernia defect reduces the risk of hernia-related bowel obstruction. Thus, the reduction in long-term risk of bowel obstruction should continue to be an integral part of the shared decision-making process when discussing surgical management of VH with patients. It is important to note that, due to the reliance on administrative coding, we are unable to determine whether bowel obstruction was caused by the hernia itself or by other factors such as adhesions. With that said, the low hazard ratio demonstrates a meaningful reduction in overall bowel obstruction risk associated with surgical repair, suggesting that repair may play a protective role regardless of the exact etiology.

Furthermore, in this analysis, the hazard for opioid misuse was comparable between the surgical cohort and the non-surgical cohort (0.97, [0.93, 1.01]) (Table 2; Fig. 1). This suggests that the impact of VHR on pain relief may be limited or incompletely understood. Chronic pain after VHR ranges from 7 to 41% [15], with presence of preoperative pain shown to be a significant predictor of postoperative chronic pain [16, 17]. The multifactorial nature of chronic pain in this patient population suggests the importance of setting realistic expectations during preoperative counseling. Additionally, studies incorporating patient-reported outcomes of both preoperative and postoperative care can provide valuable insights into patients’ experiences of pain.

The management of VH is evolving, with a major shift over the last five years. Increasingly, VH is no longer viewed as an isolated structural defect but rather a defect in a cohesive functional unit referred as the abdominal core [5–9]. ACH recognizes the interdependence of the abdominal wall, flank, diaphragm, spine, and pelvic floor [5, 6] as evidenced by the literature [18, 19]. This concept of functional, rather than purely structural, abdominal wall surgery is not new: European surgeons described the functional interdependence of the abdominal wall more than four decades ago, notably in Chevrel’s foundational text on abdominal wall surgery, in which Stoppa, Rives, and Caix developed early concepts of functional abdominal wall reconstruction [20]. The present findings extend this long-standing European surgical tradition into a large, contemporary, population-level dataset. This holistic view has important implications for the management of VH, as treating the abdominal core as an interconnected unit may improve outcomes beyond just repairing the hernia defect.

The cornerstone of this approach is the idea of regional interdependence, a concept widely discussed in physical therapy literature. This concept suggests that a patient’s primary musculoskeletal symptom may be directly or indirectly influenced by impairments in other regions of the body [21]. Research has supported this in other domains, for instance, treating hip dysfunction can alleviate impairments in the knee or addressing pain in the thoracic spine could improve neuropathic pain in the upper extremity [22–24]. When applying this framework to VH, further research is needed to evaluate how reconstructing an isolated component of the abdominal core affects the other components.

Our analysis showed that patients who underwent surgery had a higher hazard of physical therapy utilization (HR 1.76, 95% CI 1.73–1.80) (Table 2; Fig. 1). Several explanations should be considered, and we caution against reading this as straightforward evidence of a causal effect of surgery on PT engagement. First, surgical patients are, by definition, in more frequent contact with the healthcare system during the postoperative period, creating greater opportunity for referral and surveillance bias — a higher likelihood that any pre-existing or incidental need for PT is identified and coded simply because these patients are seen more often. Second, administrative claims data cannot distinguish the clinical indication for a given PT encounter; the PT utilization captured here may reflect therapy for the surgical incision or general perioperative deconditioning rather than PT specifically targeting abdominal core, spine, or pelvic floor impairment. Third, routine postoperative activity-restriction counseling and referral patterns following VHR — rather than a formalized ACH-based rehabilitation pathway — likely drive much of this utilization, and there remains an alarming lack of standardized physical therapy regimens for patients with derangements of the abdominal core [25]. Whether PT utilization after VHR reflects a cause, a consequence, or simply a correlate of closer perioperative surveillance cannot be resolved with administrative data and is a central question for prospective work with granular referral and utilization data.

The finding that VHR is associated with a modest increase in the hazard of spine and pelvic floor dysfunction (HR 1.05, 95% CI 1.02–1.07) merits particular attention rather than a peripheral mention. Under the regional interdependence framework, this is one of the few population-level signals to date suggesting that modulating one component of the abdominal core — the anterior abdominal wall — may have a measurable downstream effect on other core components, namely the spine and pelvic floor. This is consistent with prior work demonstrating that abdominal wall reconstruction is associated with changes in back pain and abdominal-wall-related quality of life [26], which supports the broader premise of interconnectedness between the abdominal wall and spine that motivates the abdominal core health framework. We therefore view this finding as one of the more clinically important observations of this study rather than an isolated or negligible signal, warranting confirmation and mechanistic exploration in future prospective work. At the same time, several alternative explanations must be weighed. Administrative datasets are limited in their ability to grade functional outcomes accurately, and detection or surveillance bias — surgical patients are monitored more closely postoperatively and are therefore more likely to have incidental musculoskeletal diagnoses coded — cannot be excluded. Unmeasured confounding, including preoperative core weakness or activity level that could independently predispose patients to both surgery and spine/pelvic floor diagnoses, may also contribute. We present this association as biologically plausible and worthy of emphasis, while stopping short of causal interpretation.

Di Stasi et al. presents the first pilot protocol for a randomized controlled trial that investigates a standardized post-operative physical therapy that utilizes ACH principles to improve function and patient-reported outcomes following VHR [8]. Integration of rehabilitation with pre- and post-operative surgical care is well-established in orthopedic surgery, where surgeons are actively involved in determining weight-bearing status, extent and timing of physical therapy, and selecting specific exercises [27]. Incorporating a similar collaborative approach in VHR could improve postoperative outcomes and recovery.

There is also literature to demonstrate that VHR may be analogous to musculoskeletal tendon repair, an area in which physical therapy plays an important role [9]. Perez et al. demonstrates that the linea alba, the structure in which the right and left fascia of the rectus abdominis muscle fuse in the midline, shares both anatomical and functional similarities to other musculoskeletal tendons. Moreover, they exhibit similar surgical principles during repair [9]. Despite these similarities, physical therapy, which is integral in management of tendon injury and repair, is not routinely implemented in VHR. Although surgical patients faced a higher likelihood of utilizing physical therapy in this study, only 16.3% did so. Future work should consider implementing principles from musculoskeletal repair into VHR.

By incorporating comprehensive management of these components post-repair, we may effectively mitigate the risk of downstream health impairments. The work by Di Stasi will help to identify relevant measures. Looking forward, we aim to establish both short- and long-term metrics to evaluate the efficacy of new protocols designed to improve these outcomes.

Several important limitations must be acknowledged in this analysis. First, an important limitation of this study is the inherent challenge in using administrative claims data to assess complex functional outcomes. Diagnoses such as spine and pelvic floor dysfunction, constipation, incontinence, and genital prolapse are frequently underreported or misclassified in billing data. The presence of an ICD-9 and − 10 diagnostic code does not confirm a validated clinical diagnosis, nor does it reflect symptom severity, duration, or impact on quality of life. We must also acknowledge that this data cannot determine whether functional disorders developed before or after VHR, or whether surgery directly contributed to their onset or improvement. Thus, the findings should be interpreted as associations, not as causal relationships.

Second, the TriNetX database lacks specific details on VH characteristics, procedures and post-operative care. Additionally, unlike databases such as the American College of Surgeons’ National Surgical Quality Improvement Program, TriNetX does not provide information on the urgency of the procedures. However, we attempted to mitigate this limitation by excluding any patients that had surgery within 5 days of VH diagnosis. We also excluded VH diagnoses associated with coding for “obstruction” or “gangrene,” as well as VHR procedure codes indicating “incarcerated or strangulated” hernias, since these typically require urgent or emergent surgery. Moreover, physical therapy utilization data may not specifically reflect therapy directed at abdominal core dysfunction, introducing potential misclassification bias. Furthermore, we were unable to account for external factors such as accidents or traumatic injuries, which may have independently prompted physical therapy and confounded our findings. Relatedly, our cohort was deliberately broad, including all VH sizes and complexities as well as both primary and incisional hernias, to maximize sample size and generalizability. This heterogeneity may obscure clinically meaningful associations: the physiologic and functional consequences of repairing a small, uncomplicated primary hernia are unlikely to be equivalent to those of repairing a large, complex incisional hernia with significant loss of domain, and abdominal core function may be differentially affected across this spectrum. Because TriNetX coding does not capture hernia size, defect area, or repair complexity (e.g., component separation versus simple primary closure), we were unable to stratify our analyses accordingly. It is possible that the modest aggregate associations we observed — particularly for spine and pelvic floor dysfunction — mask larger effects concentrated among patients undergoing more extensive reconstructions, an important question for future prospective work with granular clinical detail. We suggest that novel or existing hernia specific databases which capture defect and operative details be utilized for follow up studies that target these clinical questions.

Despite these limitations, a key strength of this study is the large sample size across numerous organizations and regions across the globe. The TriNetX database’s wide scope uses data from various healthcare organizations which enhances the generalizability of our findings. Additionally, the rigor of the time-varying analysis accounts for immortal time bias, which further improves this study.

## Conclusion

In conclusion, elective VHR was associated with a reduced hazard of bowel obstruction. However, no significant associations were observed between surgery and opioid misuse or most functional disorders. While a higher hazard of physical therapy utilization was seen among surgical patients, which may reflect increased healthcare engagement or routine postoperative care, rather than improved or worsened core function. Importantly, the associations observed for spine and pelvic dysfunction, constipation, incontinence, and genital prolapse must be interpreted cautiously, as these outcomes may represent pre-existing or co-occurring conditions rather than consequences of surgery.

These findings highlight the need for future prospective studies with clinically validated outcomes to better understand the impact of hernia repair on abdominal core function. Multidisciplinary postoperative care, including individualized physical therapy, may play an important role in optimizing recovery and addressing persistent symptoms. Clear communication with patients about expected outcomes, including the potential for chronic pain and unclear impact on functional disorders, is essential during shared decision-making.

## Supplementary Information

Below is the link to the electronic supplementary material.

Supplementary Material 1

## Data Availability

Data are available upon reasonable request
