# Supplementary material for: Operative vs non-operative management of ventral hernia: a population based study of long-term benefits and consequences
Source: Hernia. 2026 Sep 16;30(1):352. doi: 10.1007/s10029-026-03845-x (PMC13582302; doi:10.1007/s10029-026-03845-x)
Supplement: Supplementary file 1 [file 10029_2026_3845_MOESM1_ESM.docx]

Supplemental Table 1. ICD-9 and ICD-10 Codes used to determine Ventral Hernia Diagnosis and ICD-PCS and CPT Codes used to determine Ventral Hernia Repair

| **ICD10** | **ICD10 Name** | **ICD9** | **ICD9 Name** |
| --- | --- | --- | --- |
| **Codes for Ventral Hernia Diagnosis** | | | |
| K42.9 | Umbilical hernia without obstruction or gangrene | 553.10 | Umbilical hernia without mention of obstruction or gangrene |
| K43.2 | Incisional hernia without obstruction or gangrene | 553.21 | Incisional hernia without mention of obstruction or gangrene |
| K43.9 | Ventral hernia without obstruction or gangrene | 553.20 | Ventral, unspecified, hernia without mention of obstruction or gangrene |
|  |  | 553.29 | Other ventral hernia without mention of obstruction or gangrene |
| **ICD-PCS** | **ICD-PCS Name** | **CPT** | **CPT Name** |
| **Codes for Ventral Hernia Repair** | | | |
| 0WQF0ZZ | Repair Abdominal Wall, Open Approach | 49590 | Repair Spigelian Hernia |
| 0WQF3ZZ | Repair Abdominal Wall, Percutaneous Approach | 49585 | Repair Umbilical Hernia, age 5 years or older; reducible |
| 0WQF4ZZ | Repair Abdominal Wall, Percutaneous Endoscopic Approach | 49570 | Repair Epigastric Hernia; reducible |
| 0WQFXZZ | Repair Abdominal Wall, External Approach | 49560 | Repair Initial Incisional or Ventral Hernia; reducible |
| 0WUF07Z | Supplement Abdominal Wall with Autologous Tissue Substitute, Open Approach | 49565 | Repair Recurrent Incisional or Ventral Hernia; reducible |
| 0WUF0JZ | Supplement Abdominal Wall with Synthetic Substitute, Open Approach | 49652 | Laparoscopy, surgical, repair, ventral, umbilical, spigelian or epigastric hernia (includes mesh insertion, when performed); reducible |
| 0WUF0KZ | Supplement Abdominal Wall with Nonautologous Tissue Substitute, Open Approach | 49654 | Laparoscopy, surgical, repair, incisional hernia (includes mesh insertion, when performed); reducible |
| 0WUF47Z | Supplement Abdominal Wall with Autologous Tissue Substitute, Percutaneous Endoscopic Approach | 49656 | Laparoscopy, surgical, repair, recurrent incisional hernia (includes mesh insertion, when performed); reducible |
| 0WUF4JZ | Supplement Abdominal Wall with Synthetic Substitute, Percutaneous Endoscopic Approach | 49591 | Repair of anterior abdominal hernia(s) (i.e., epigastric incisional, ventral, umbilical, spigelian), any approach (i.e., open, laparoscopic, robotic), initial, including placement of mesh or other prosthesis, when performed, total length of defect(s); less than 3 cm, reducible |
| 0WUF4KZ | Supplement Abdominal Wall with Nonautologous Tissue Substitute, Percutaneous Endoscopic Approach | 49593 | 3 cm to 10 cm, reducible |
|  |  | 49595 | Greater than 10 cm, reducible |
|  |  | 49613 | Repair of anterior abdominal hernia(s) (i.e., epigastric incisional, ventral, umbilical, spigelian), any approach (i.e., open, laparoscopic, robotic), recurrent, including placement of mesh or other prosthesis, when performed, total length of defect(s); less than 3 cm, reducible |
|  |  | 49615 | 3 cm to 10 cm, reducible |
|  |  | 49617 | Greater than 10 cm, reducible |

Supplemental Table 2. ICD-9, ICD-10, and CPT Codes for Selection of Outcomes

| **ICD10** | **ICD10 Name** | **ICD9** | **ICD9 Name** |
| --- | --- | --- | --- |
| **Opioid Misuse** | | | |
| Z79.891 | Long term (current) use of opiate analgesic | V58.69 | Long-term (current) use of other medications |
| F11.10 | Opioid abuse, uncomplicated | 305.5 | Opioid abuse, unspecified |
|  |  | 305.51 | Opioid abuse, continuous |
|  |  | 305.52 | Opioid abuse, episodic |
|  |  | 305.53 | Opioid abuse, in remission |
| T40.2 | Poisoning by, adverse effect of and underdosing of other opioids | 965.09 | Poisoning by other opiates and related narcotics |
|  |  | E85.02 | Accidental poisoning by other opiates and related narcotics |
| T40.69 | Poisoning by, adverse effect of and underdosing of other narcotics | 965.09 | Poisoning by other opiates and related narcotics |
|  |  | E85.02 | Accidental poisoning by other opiates and related narcotics |
| T40.0 | Poisoning by, adverse effect of and underdosing of opium | 965.00 | Poisoning by opium (alkaloids), unspecified |
|  |  | E85.02 | Accidental poisoning by other opiates and related narcotics |
| T39 | Poisoning by, adverse effect of and underdosing of nonopioid | 965.4 | Poisoning by aromatic analgesics, not elsewhere classified |
|  |  | 965.5 | Poisoning by pyrazole derivatives |
|  |  | 965.6 | Poisoning by antirheumatics (antiphlogistics) |
|  |  | E85.04 | Accidental poisoning by aromatic analgesics, not elsewhere classified |
| T40.1 | Poisoning by and adverse effect of heroin | 965.01 | Poisoning by heroin |
|  |  | E85.00 | Accidental poisoning by heroin |
| T40.3 | Poisoning by, adverse effect of and underdosing of methadone | 965.02 | Poisoning by Methadone |
|  |  | E85.01 | Accidental poisoning by methadone |
| T40.5 | Poisoning by, adverse effect of and underdosing of cocaine | 970.81 | Poisoning by cocaine |
|  |  | E85.42 | Accidental poisoning by psychostimulants |
| **Obstruction** | | | |
| K56.609 | Unspecified intestinal obstruction, unspecified as to partial versus complete obstruction | 560.90 | Unspecified intestinal obstruction |
| K42.0 | Umbilical hernia with obstruction, without gangrene | 552.1 | Umbilical hernia with obstruction |
| K43.0 | Incisional hernia with obstruction, without gangrene | 552.21 | Incisional hernia with obstruction |
| K43.6 | Other and unspecified ventral hernia with obstruction, without gangrene | 552.20 | Ventral, unspecified, hernia with obstruction |
|  |  | 552.29 | Other ventral hernia with obstruction |
| K46.0 | Unspecified abdominal hernia with obstruction, without gangrene | 552.90 | Hernia of unspecified site, with obstruction |
| **Spine and Pelvic Floor Diagnosis** | | | |
| M54.5 | Low Back Pain | 724.2 | Lumbago |
| M54.16 | Radiculopathy, lumbar region | 724.4 | Thoracic or lumbosacral neuritis or radiculitis unspecified |
| M54.17 | Radiculopathy, lumbosacral region | 724.4 | Thoracic or lumbosacral neuritis or radiculitis unspecified |
| M54.4 | Lumbago with sciatica | 724.3 | Sciatica |
| M51.9 | Thoracic, thoracolumbar, and lumbosacral intervertebral disc | 722.2 | Displacement of intervertebral disc site unspecified without myelopathy |
|  |  | 722.3 | Schmorl's nodes of unspecified region |
|  |  | 722.39 | Schmorl's nodes of other spinal region |
|  |  | 722.7 | Intervertebral disc disorder with myelopathy unspecified region |
|  |  | 722.90 | Other and unspecified disc disorder of unspecified region |
| R10.2 | Pelvic and perineal pain | 789.09 | Abdominal pain other specified site |
|  |  | 625.9 | Unspecified symptom associated with female genital organs |
|  |  | 608.9 | Unspecified disorder of male genital organs |
| S89.81XA | Other specified injuries of right lower leg, initial encounter | 908.9 | Late effect of unspecified injury |
|  |  | 959.7 | Knee, leg, ankle, and foot injury |
|  |  | V58.89 | Other specified aftercare |
| S89.82XA | Other specified injuries of left lower leg, initial encounter | 908.9 | Late effect of unspecified injury |
|  |  | 959.7 | Knee, leg, ankle, and foot injury |
|  |  | V58.89 | Other specified aftercare |
| M99.05 | Segmental and somatic dysfunction of pelvic region | 739.5 | Nonallopathic lesions of pelvic region not elsewhere classified |
| M99.04 | Segmental and somatic dysfunction of sacral region | 739.4 | Nonallopathic lesions of sacral region not elsewhere classified |
| M99.03 | Segmental and somatic dysfunction of lumbar region | 739.3 | Nonallopathic lesions of lumbar region not elsewhere classified |
| **Constipation** | | | |
| K59.00 | Constipation | 564.00 | Constipation, unspecified |
| K59.02 | Outlet dysfunction constipation | 564.02 | Outlet dysfunction constipation |
| K59.00 | Constipation, unspecified | 564.00 | Constipation, unspecified |
| K59.09 | Other constipation | 564.09 | Other constipation |
| **Incontinence** | | | |
| R32 | Unspecified urinary incontinence | 788.30 | Urinary incontinence, unspecified |
| N39.3 | Stress incontinence (female) (male) | 625.60 | Stress incontinence, female |
|  |  | 788.32 | Stress incontinence, male |
| N39.41 | Urge incontinence | 788.31 | Urge incontinence |
| N39.46 | Mixed incontinence | 788.33 | Mixed incontinence (male) (female) |
| **Prolapse** | | | |
| N81.9 | Female genital prolapse, unspecified | 618.00 | Unspecified prolapse of vaginal walls |
| N81.8 | Other female genital prolapse | 618.8 | Other specified genital prolapse |
| **CPT** | **CPT Name** |  |  |
| **Physical Therapy Utilization** | | | |
| 97161 | Physical therapy evaluation: low complexity |  |  |
| 97162 | Physical therapy evaluation: moderate complexity |  |  |
| 97163 | Physical therapy evaluation: high complexity |  |  |

Supplemental Table 3. ICD-9 and ICD-10 Codes for Covariates

| **ICD10** | **ICD10 Name** | **ICD9** | **ICD9 Name** |
| --- | --- | --- | --- |
| **Obesity** | | | |
| E66.0 | Obesity due to Excess Calories |  |  |
| E66.01 | Morbid (severe) Obesity due to Excess Calories | 278.01 | Morbid Obesity |
| E66.09 | Other Obesity due to Excess Calories |  |  |
| E66.1 | Drug-Induced Obesity |  |  |
| E66.2 | Morbid (severe) Obesity with Alveolar Hypoventilation | 278.02 | Obesity Hypoventilation Syndrome |
| E66.8 | Other Obesity | 278.03 | Other Specified Obesity |
| E66.9 | Obesity, Unspecified | 278.00 | Obesity, Unspecified |
| **Smoking Status** | | | |
| F17 | Used for Nicotine Dependence | 305.1 | Tobacco Use Disorder |
| **Diabetes Mellitus** | | | |
| E10 | Type 1 Diabetes | 249.00 | Secondary Diabetes Mellitus |
| E11 | Type 2 Diabetes | 250.0-92 | Diabetes Mellitus |
| **Chronic Obstructive Pulmonary Disease** | | | |
| J44 | Other Chronic Obstructive Pulmonary Disease | 491 | Chronic Bronchitis |
|  |  | 492 | Emphysema |
|  |  | 496 | Chronic Airway Obstruction, Not Elsewhere Classified |
| **Hypertension** | | | |
| I10 | Essential (primary) hypertension | 401 | Essential Hypertension |
|  |  | 402 | Hypertensive Heart Disease |
|  |  | 403 | Hypertensive Renal Disease |
|  |  | 404 | Hypertensive Heart and Renal Disease |
|  |  | 405 | Secondary Hypertension |
| **Neoplasm** | | | |
| C00-C96 | Malignant Neoplasm | 140-208 | Malignant Neoplasm |
| **Congenital Malformations and Deformations** | | | |
| Q00-Q99 | Congenital Malformations | 740-759 | Congenital Malformations |
